# Supplementary material for: The ALT pathway generates telomere fusions that can be detected in the blood of cancer patients
Source: Nat Commun. 2024 Jan 2;15:82. doi: 10.1038/s41467-023-44287-8 (PMC10762111; doi:10.1038/s41467-023-44287-8)
Supplement: Supplementary file 1 — Supplementary Information [file 41467_2023_44287_MOESM1_ESM.pdf]

Supplementary Figures

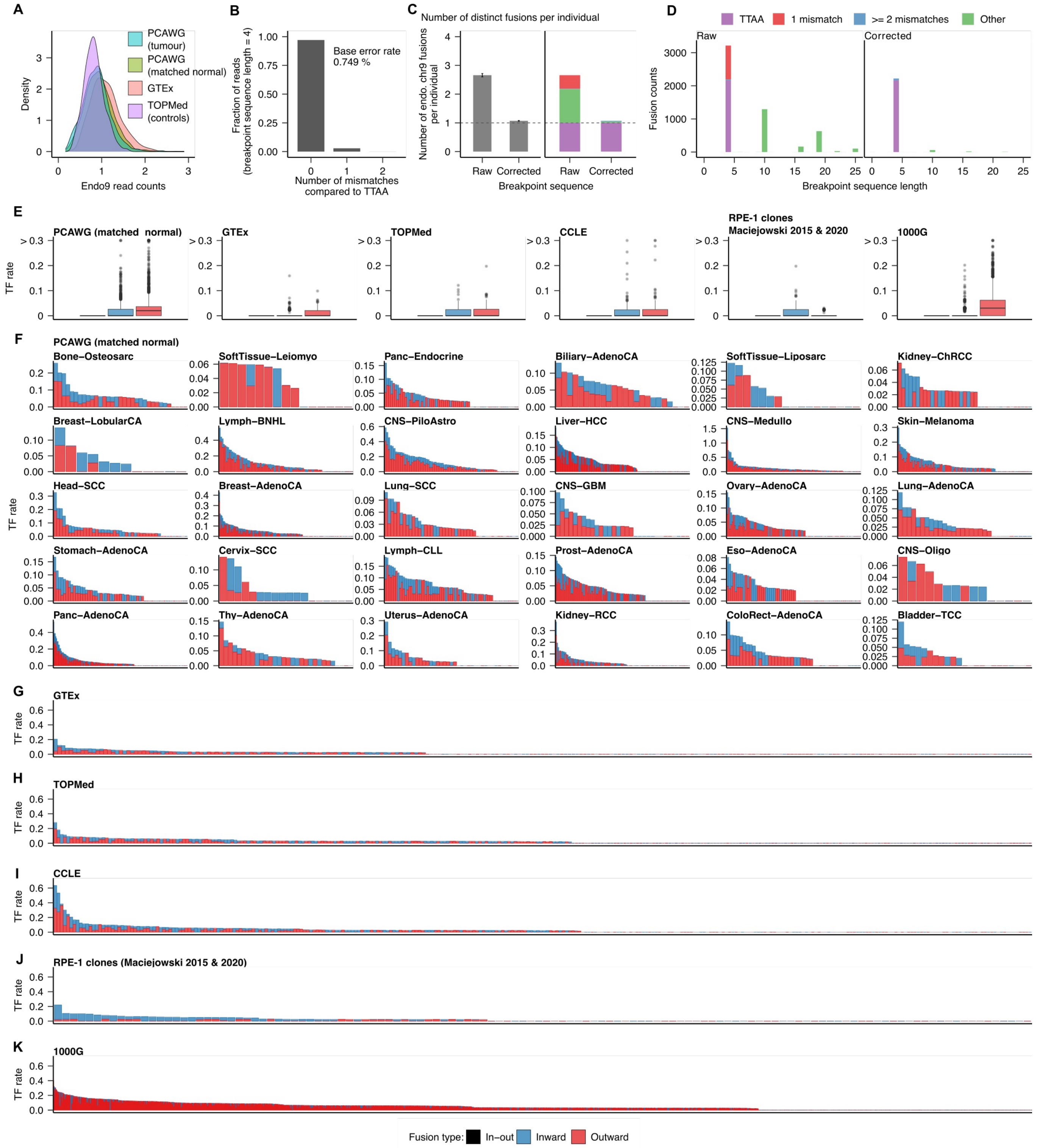

Supplementary Figure 1. (A) Distribution of the rate of endogenous chromosome 9 fusions detected in PCAWG tumour and matched-normal samples, and healthy blood samples from GTEx and TOPMed. Overall, the distribution is comparable across projects, indicating that TF rates enable analysis across samples sequenced using different read lengths and at variable sequencing depth. **(B)** Fraction of reads mapping to the endogenous chromosome 9 fusion. The x-axis corresponds to the number of mismatches between the breakpoint sequence and the expected sequence, TTAA. This analysis revealed an error rate in reads mapping to the chromosome 9 endogenous fusion of 0.749%, which is comparable to the expected error rate for Illumina sequencing. **(C)** Number of distinct chromosome 9 endogenous fusions (as determined by the breakpoint sequence) found per individual before and after error correcting the breakpoint sequences (STAR Methods). The bars indicate the fraction of reads with the expected breakpoint sequence TTAA (shown in purple), one mismatch (red), and two mismatches (blue). Only reads with a fusion breakpoint sequence of 4bp are included in this plot. As expected, we detected one breakpoint sequence at the chromosome 9 endogenous fusion after error correction in most samples. **(D)** Length of the breakpoint sequences for the endogenous chromosome 9 fusion detected per individual before and after error correction. As expected, only one breakpoint sequence of 4bp in length (TTAA) is detected in most samples. **(E)** Distribution of circular, inward and outward ALT-TFs across all samples. ALT-TF rates detected in matched-normal samples from PCAWG (**F**), GTEx (**G**), TOPMed (**h**), cancer cell lines (CCLE) (**i**), RPE-1 cells (**J**), and EBV-immortalized B cell lines from the 1000 Genome Project (**K**).

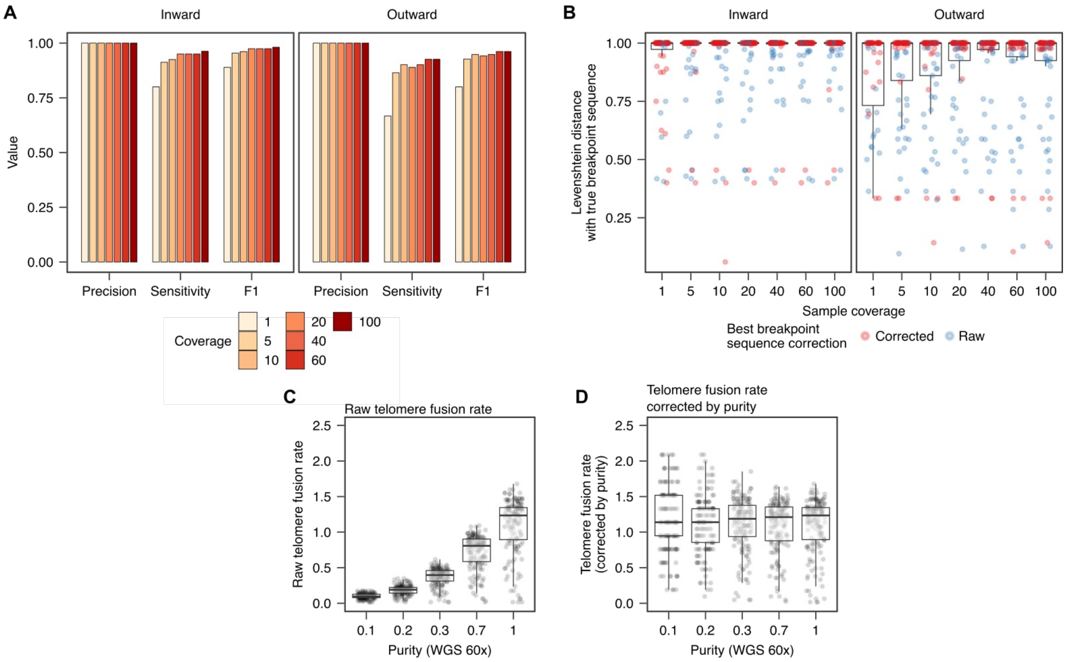

Supplementary Figure 2. Benchmarking analysis of TelFusDetector using simulations. (**A**) Performance of TelFusDetector across different depths of coverage for inward and outward ALT-TFs separately. (**B**) Levenshtein distance between the expected and observed breakpoint sequences of the ALT-TFs detected by TelFusDetector. (**C**) ALT-TF rate estimated using simulated samples across increasingly higher values of simulated tumour cellularity (or purity) and without correcting for tumour purity. (**D**) ALT-TF rates estimated for the same samples shown in **C** after correcting for purity. Note that the ALT-TF rate is the same in all samples, which is only correctly estimated after correcting for purity. All box plots show the median, first and third quartiles (boxes), and the whiskers encompass observations within 1.5x the interquartile range from the first and third quartiles.

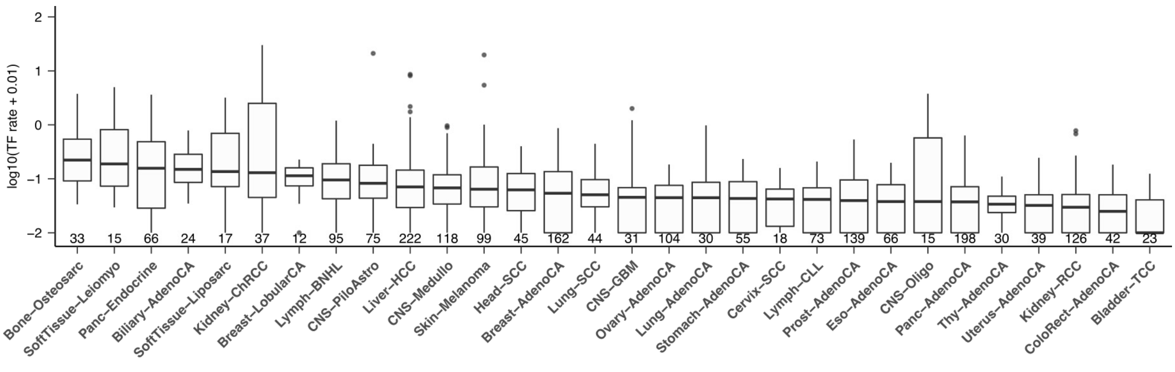

Supplementary Figure 3. (**A**) TF rates across cancer types in the PCAWG cohort. Box plots show the median, first and third quartiles (boxes), and the whiskers encompass observations within a distance of 1.5x the interquartile range from the first and third quartiles.

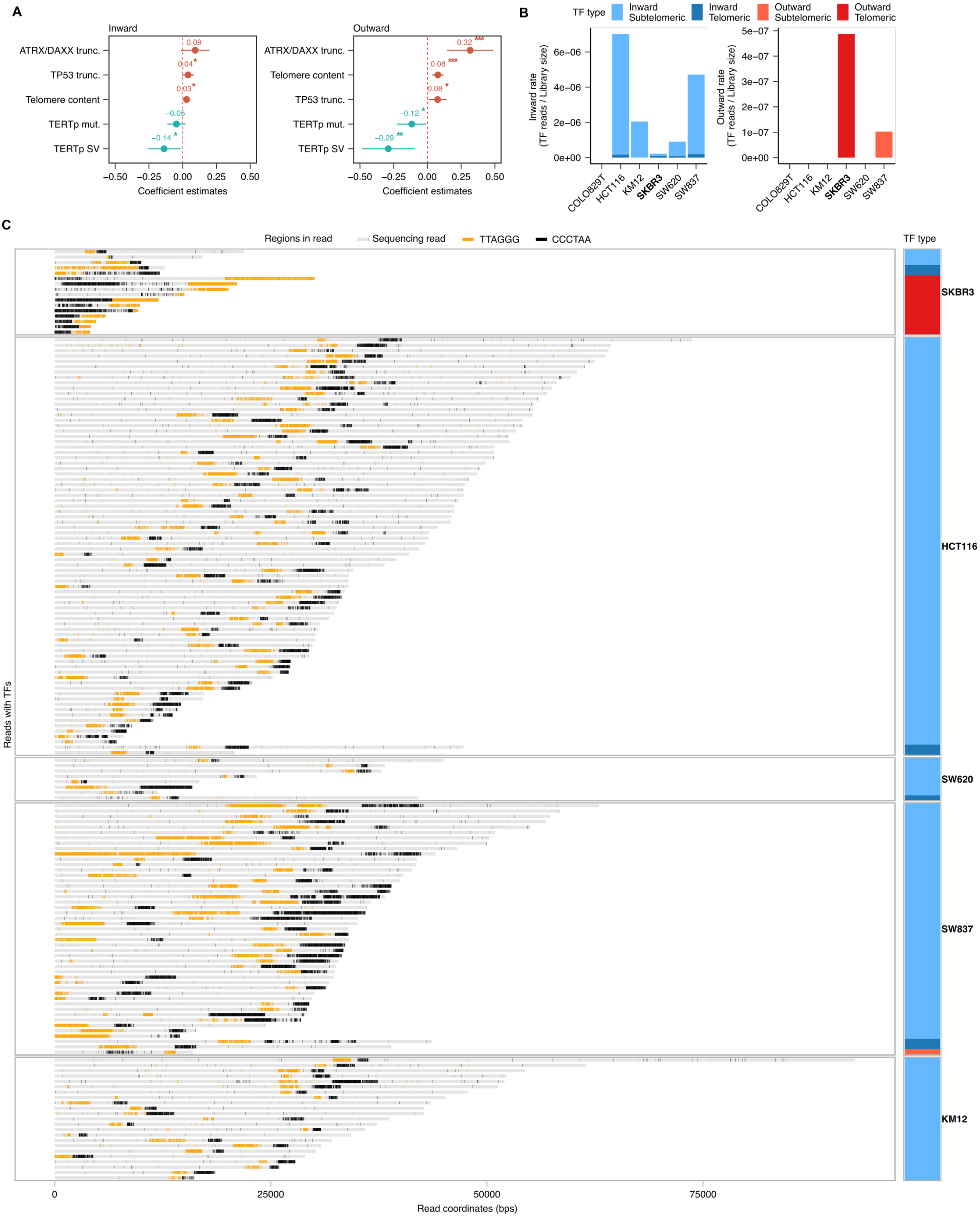

Supplementary Figure 4. (**A**) Coefficient values estimated using linear regression analysis and variable selection for the covariates with the strongest positive and negative association with TF rates. For this analysis we used the ALT status classification reported by Sieverling et al. 2020 (see also Figure 2a). (**B**) TF rates normalised by the total library size obtained for cell lines analysed using long-read sequencing technologies. The rate of outward ALT-TFs is significantly higher in the breast cancer cell line *SKBR3*, which is a used model of ALT. ALT-TFs were classified as subtelomeric if the genomic regions flanking the telomeric repeats could be unambiguously mapped to a subtelomeric region. (**C**) Schematic representation of the long reads containing ALT-TF fusions. The bar on the right represents the classification of the different fusions as described in (**B**). Intra-read telomeric repeats TTAGGG and CCCTAA are shown in orange and black, respectively.

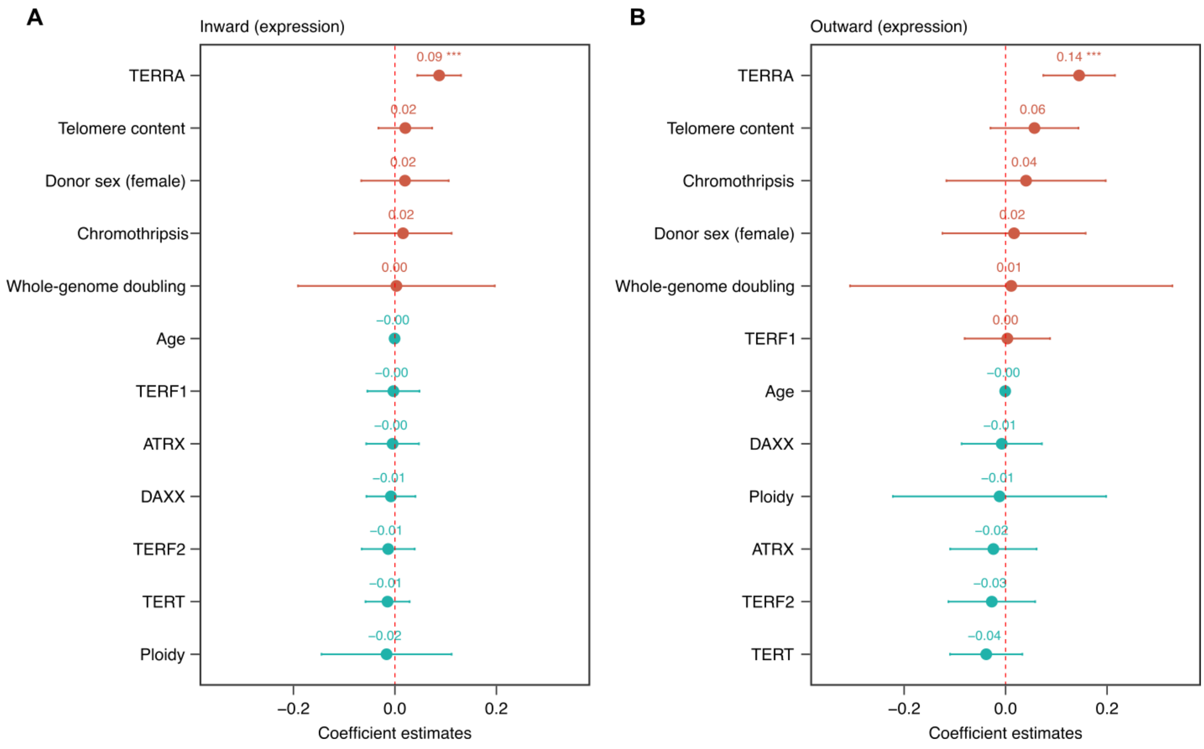

Supplementary Figure 5. Coefficient values estimated using linear regression analysis and variable selection for the covariates (levels of expression per gene) with the strongest positive and negative association with ALT-TF rates for (**A**) inward and (**B**) outward ALT-TFs. Only PCAWG tumour samples with RNA-seq data available were included in this analysis. *$P < 0.05$, **$P < 0.01$; ***$P < 0.001$.

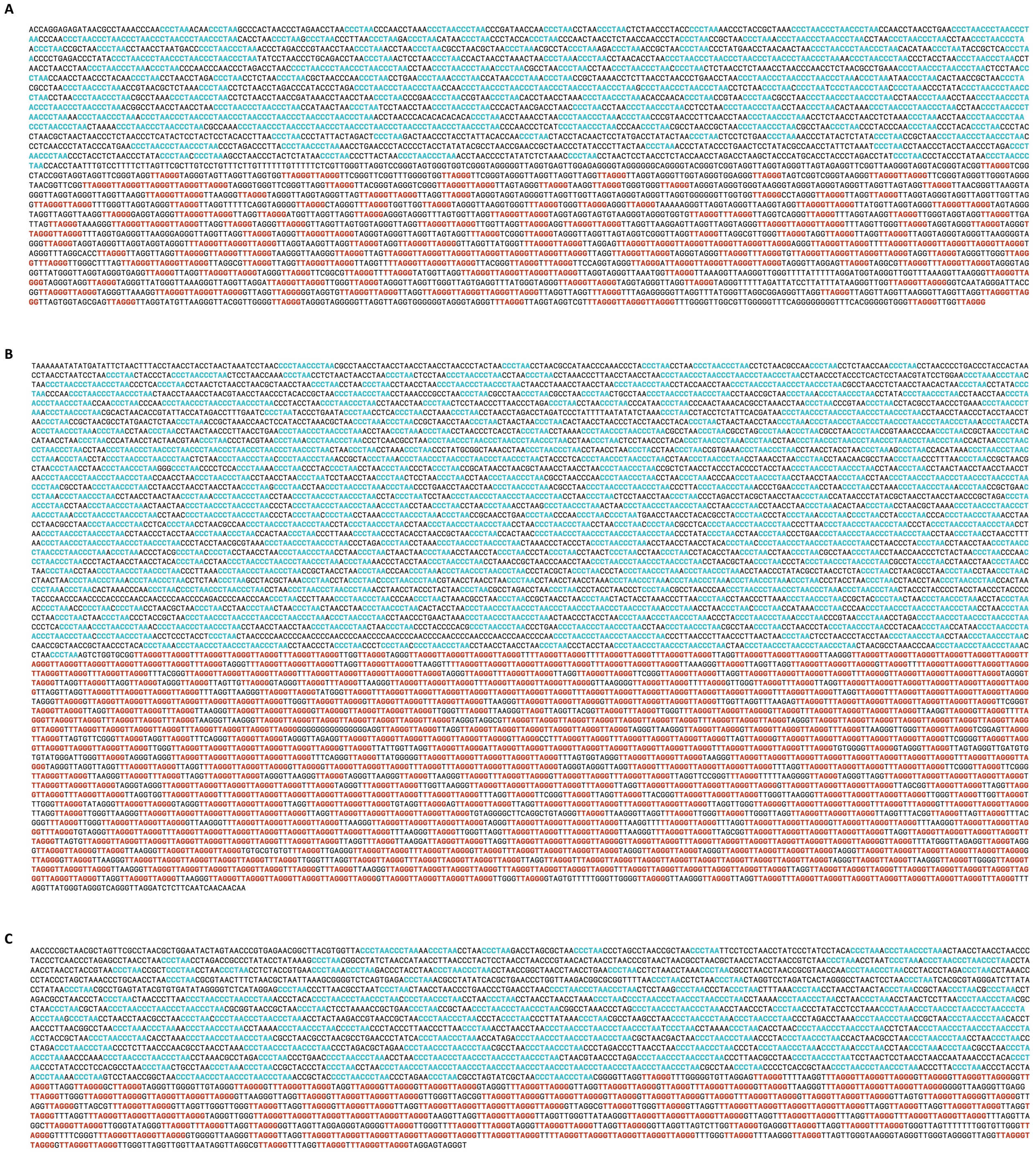

Supplementary Figure 6. (A-C) Representative examples of outward fusions detected in PacBio long-read sequencing data from the breast cancer cell line SKBR3. Telomeric repeats TTAGGG and CCCTAA are shown in red and blue, respectively.

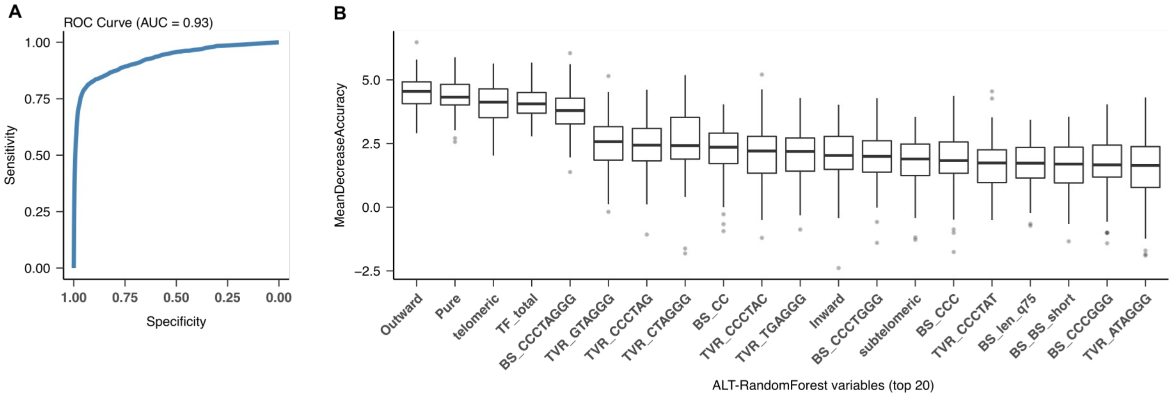

Supplementary Figure 7. Predictive power of the Random Forest models trained to predict the ALT status of tumours based on the features of ALT-TFs. **(A)** Area Under the Curve (AUC) for the Random Forest model. The results across 100 bootstrap resamples are shown. **(B)** Mean decrease in accuracy obtained for the variables used in the Random Forest model. Only the top 20 predictive features are shown in the plot. The higher the value the stronger the predictive power of each variable. Box plots show the median, first and third quartiles (boxes), and the whiskers encompass observations within a distance of 1.5x the interquartile range from the first and third quartiles. *Pure* refers to the rate of ALT-TFs with breakpoint sequences in the set of all possible circular permutations of TTAGGG and CCCTAA, whereas *Telomeric* refers to the rate of all ALT-TFs excluding those whose supporting reads map to subtelomeric regions with mapping quality (MAPQ) > 8. Covariates with the *TVR prefix* refer to the telomere variant repeat (TVR) fractions found in each sample. Covariates with the *BS* prefix refer to the rate of ALT-TFs with a specific breakpoint sequence (BS).

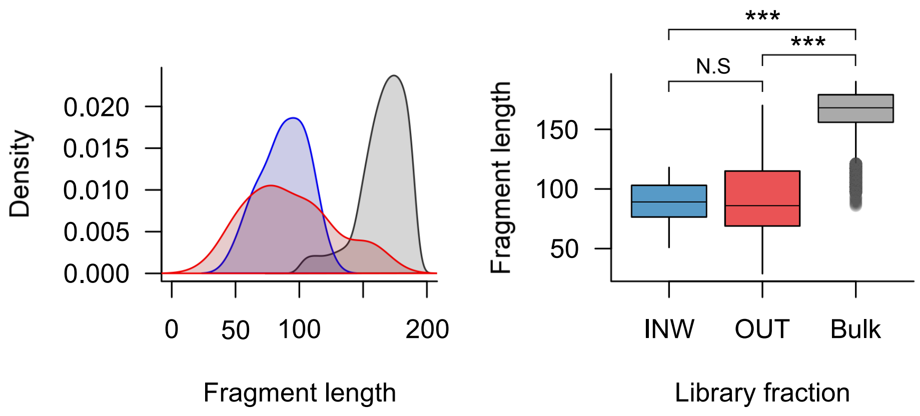

Supplementary Figure 8. Fragment size distribution for assembled fragments in ALaP-Seq libraries from mESCs with peroxidase corresponding to read pairs mapping outside telomeric regions, and read pairs containing either inward or outward fusions. The distribution of assembled fragment lengths in complete libraries has a maximum of around 180 bp, while those for inward and outward ALT-TF-containing fractions have maximum sizes below 100 bp (***$P < 0.001$; Wilcoxon rank-sum test).

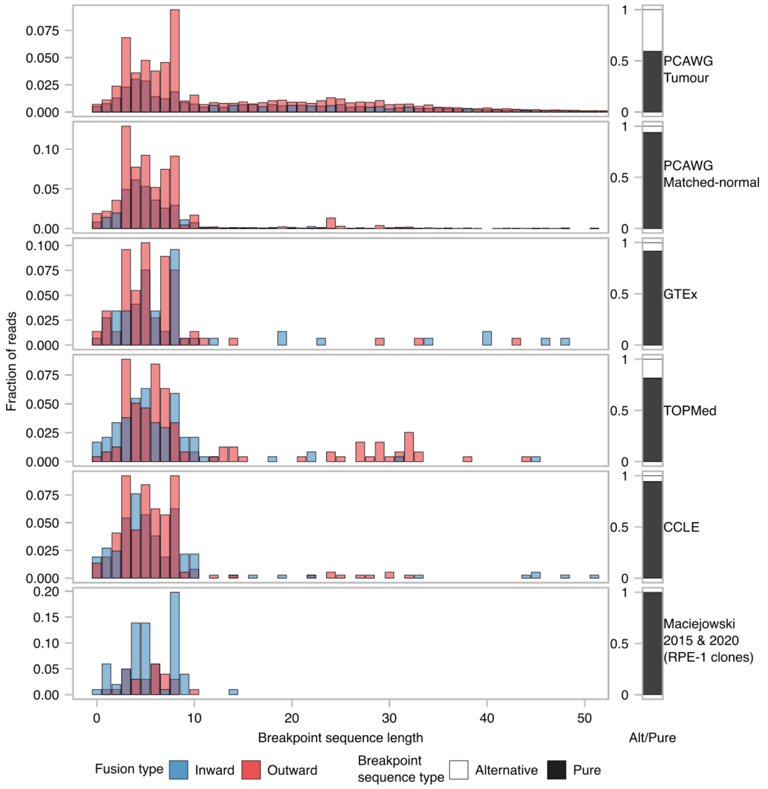

Supplementary Figure 9. Breakpoint sequence length distribution. Inward and outward fusions are shown in blue and red, respectively. The bars on the right show the fraction of ALT-TFs classified as pure (black) or alternative (white).

Inward

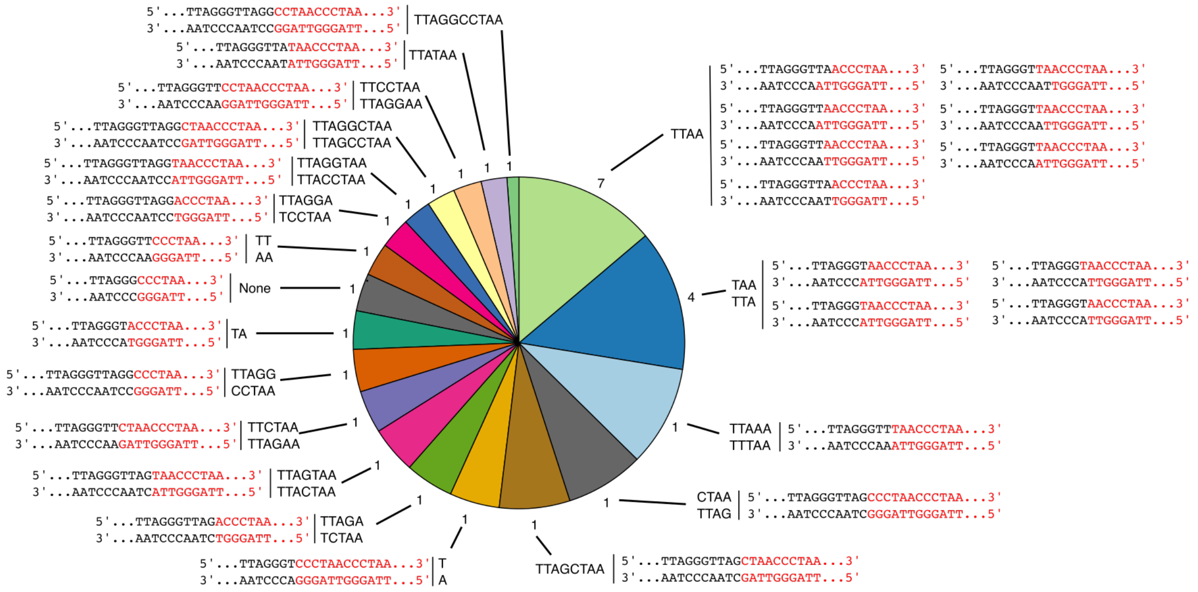

Outward

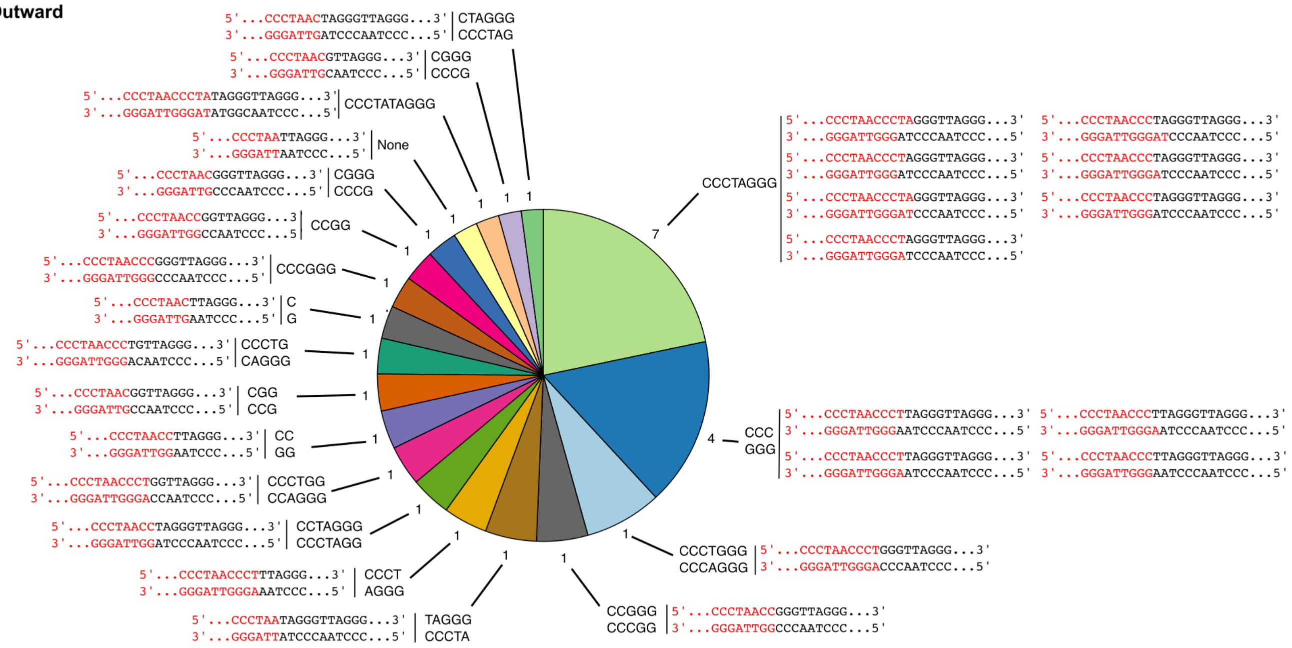

Supplementary Figure 10. Extended pie chart showing the distribution of the distinct breakpoint sequences observed in pure ALT-TFs in PCAWG tumours. The numbers around the pie charts represent the number of combinations of circular permutations of telomeric repeat motifs that can generate each breakpoint sequence. The legend reports the breakpoint sequences in both strands unless they are identical (e.g., TTAA).

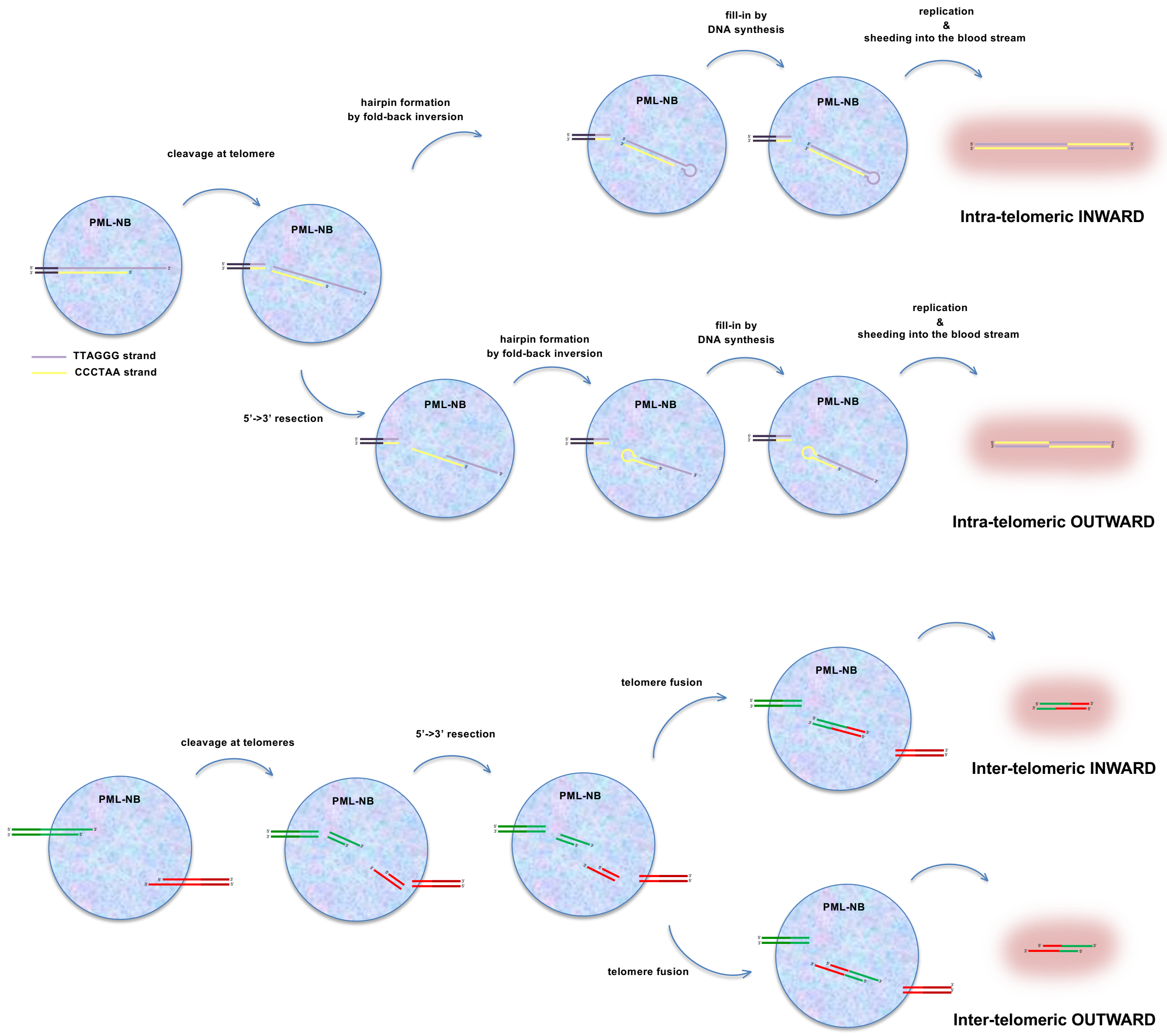

Supplementary Figure 11. Intra- and inter-telomeric ligation mechanisms in PML-NBs can lead to the formation of ALT-TF detected in the blood of cancer patients. See main text for details.

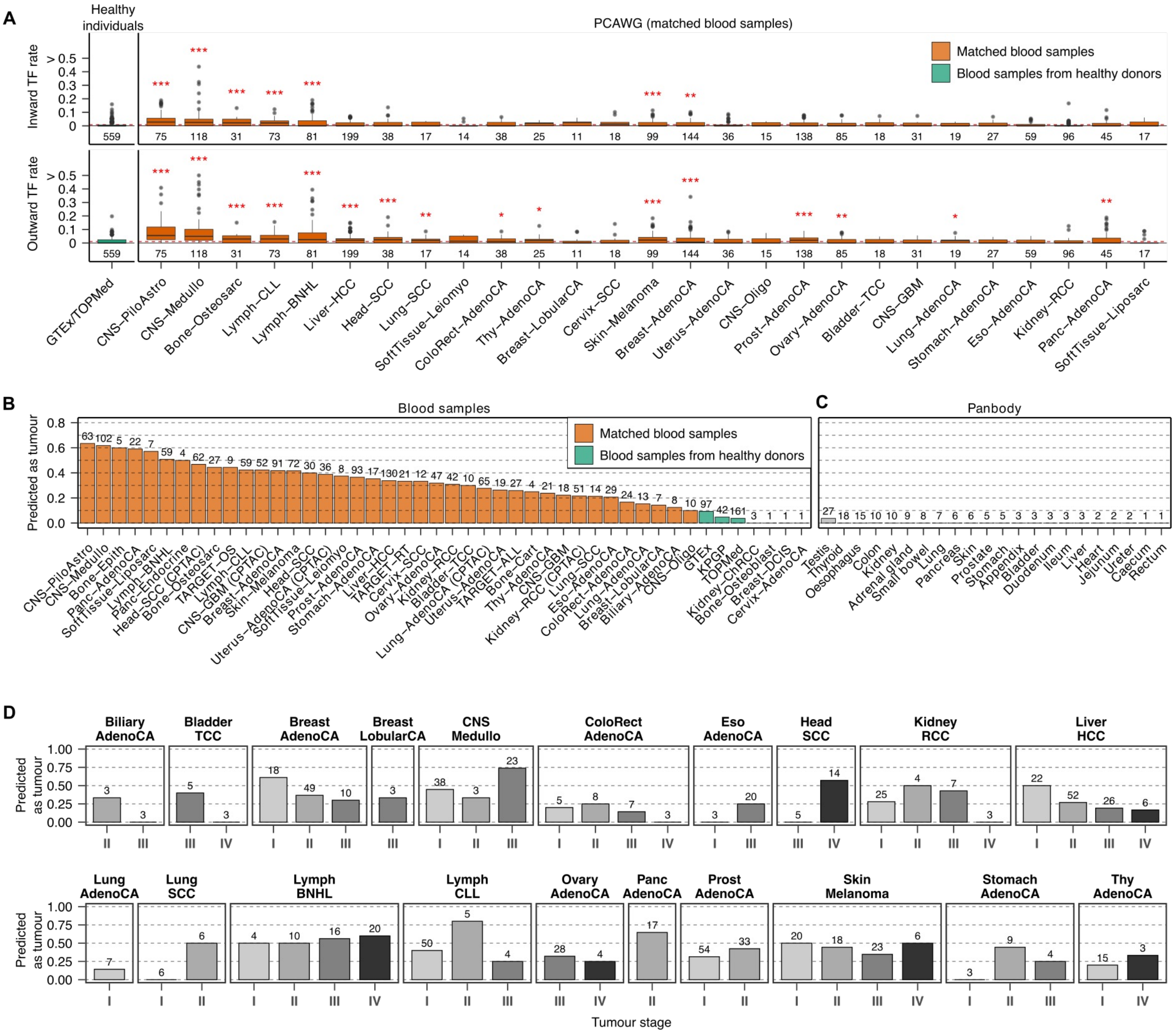

Supplementary Figure 12. (A) Rates of inward and outward ALT-TFs detected in the blood samples from healthy individuals from GTEx and TOPMed (green) and matched blood samples from PCAWG (orange). **(B)** Fraction of individuals with at least 1 ALT-TF in blood predicted to have cancer. **c**, Fraction of histologically normal samples from the Panbody study with at least 1 ALT-TF predicted as cancer samples using the same model. (**D**) Fraction of PCAWG cases with at least 1 ALT-TF in blood correctly classified as having cancer stratified according to cancer stage. Predictions in **(B-D)** were computed using 100 Random Forest models trained on features of the ALT-TFs detected in the WGS data from the matched blood samples from PCAWG, CTPAC and TARGET, and blood samples from GTEx, KPGP and TOPMed, which were used as controls. Only samples with at least 1 ALT-TF in blood were used for training. The numbers below the boxplots in **(A)** and above the bars in (**B-C)** indicate the total number of samples in each group. Box plots in **(A)** show the median, first and third quartiles (boxes), and the whiskers encompass observations within a distance of 1.5x the interquartile range from the first and third quartiles. ***$P < 0.001$; **$P < 0.01$; *$P < 0.05$, Wilcoxon rank-sum test.

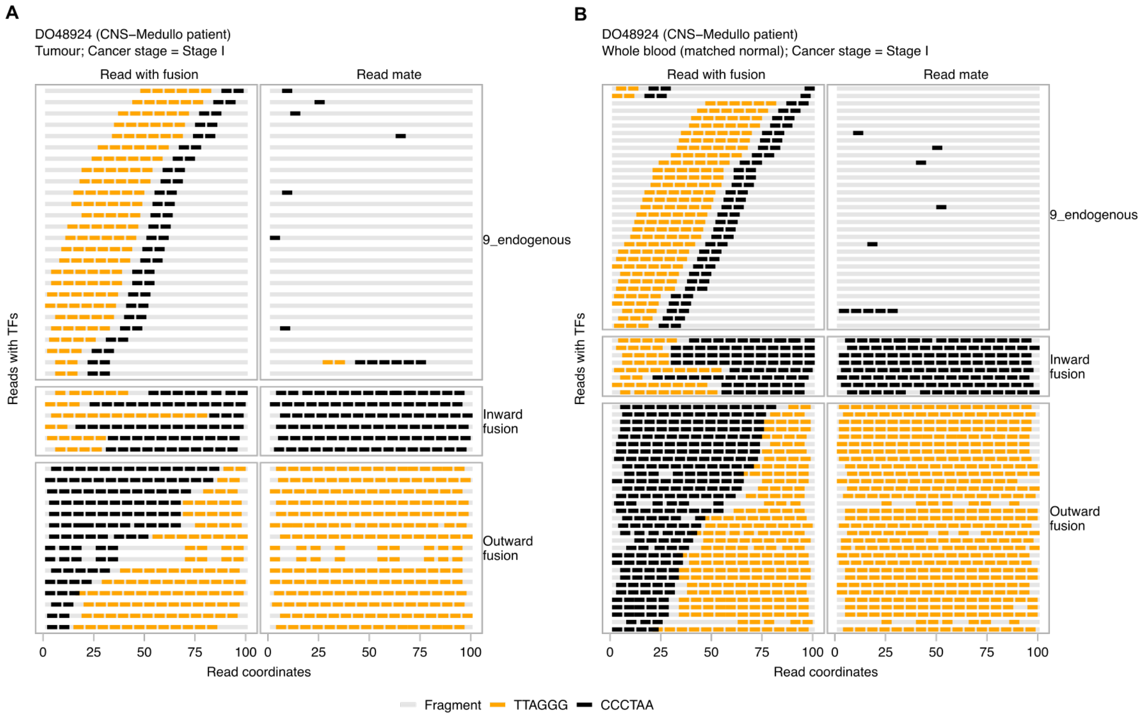

Supplementary Figure 13. Schematic representation of sequencing reads containing ALT-TFs in a medulloblastoma patient (stage I). Representation of telomeric repeats in sequencing reads containing ALT-TFs in the tumour sample (**A**) and in whole blood (**B**). Intra-read telomeric repeats TTAGGG and CCCTAA are shown in orange and black, respectively.

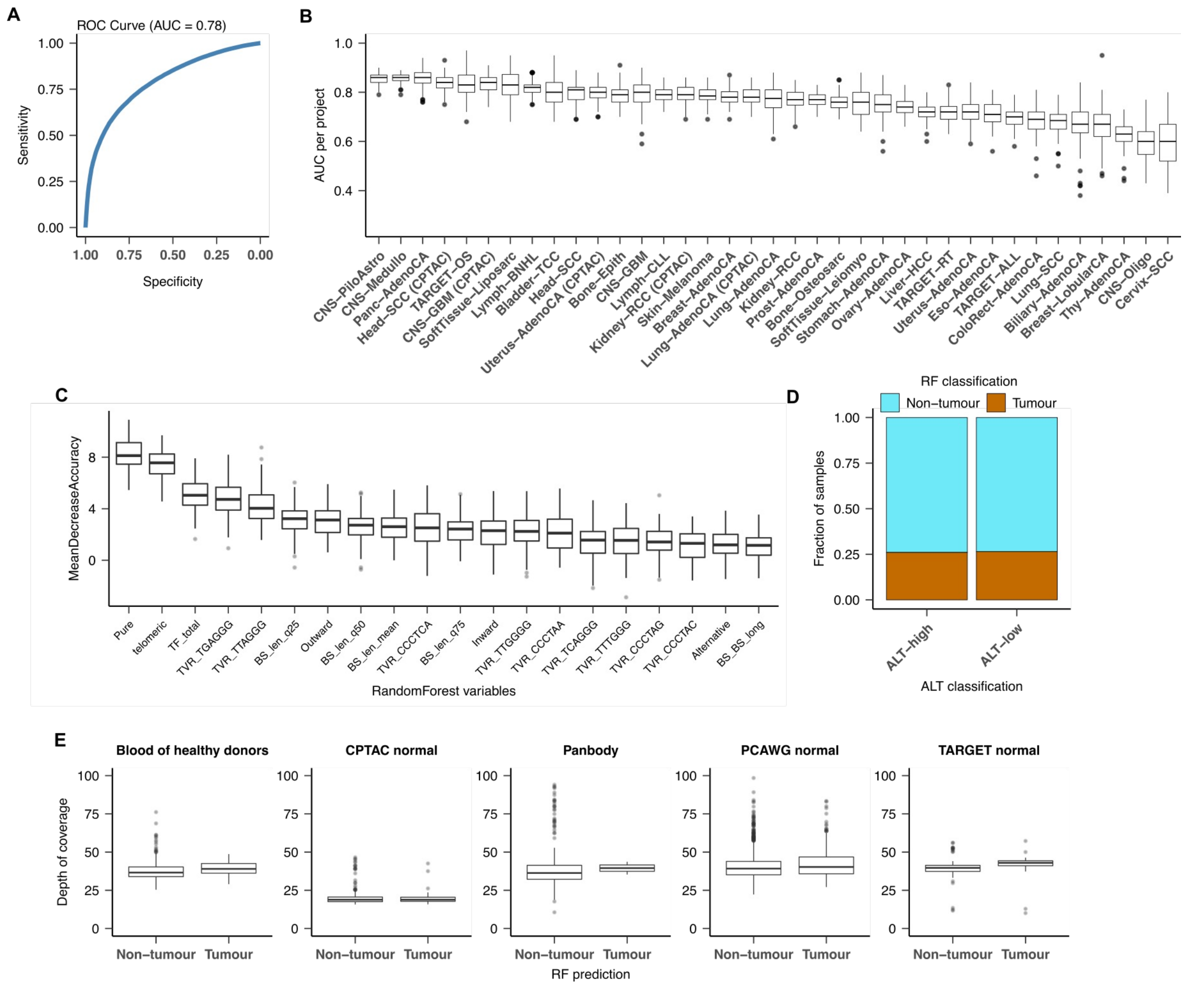

Supplementary Figure 14. Predictive power of the Random Forest models generated to predict the probability that an individual has cancer on the basis on the features of the ALT-TFs detected in blood. **(A)** Global AUC of the Random Forest model. The results across 100 bootstraps are shown. **(B)** AUC values split by cancer type. **(C)** Mean decrease in accuracy (importance) obtained for each variable used in the Random Forest model. The top 20 predictive features are shown. **(D)** Fraction of whole blood samples from the PCAWG cohort predicted as originating from a cancer patient ("Tumour") and grouped by ALT classification status. **(E)** Depth of coverage of samples predicted to belong to a cancer patient (represented by "Tumour") or not ("Non-Tumour"). Overall, this analysis indicates that the predictive power of the algorithm is not affected by the sequencing depth. Box plots show the median, first and third quartiles (boxes), and the whiskers encompass observations within a distance of 1.5x the interquartile range from the first and third quartiles.

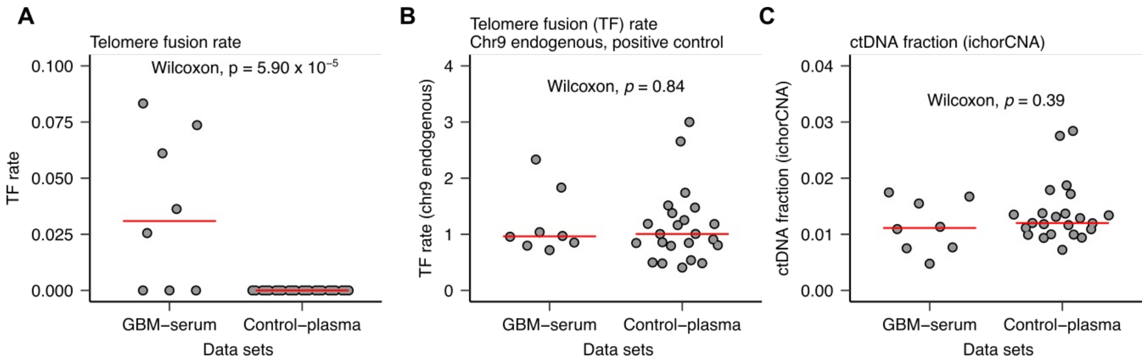

Supplementary Figure 15. (**A**) Comparison of ALT-TF rates detected in cell-free DNA from serum from glioblastoma (GBM) patients and cell-free DNA from control plasma samples. (**B**) Rate of chromosome 9 endogenous fusions in serum from GBM patients and plasma controls. (**C**) Comparison of the tumour fraction estimated using ichorCNA for the serum samples from GBM patients and plasma controls. The red bar represents the median. *P* values were computed using two-sided Wilcoxon tests.

Supplementary Tables

Supplementary Table 1. The coordinates for the endogenous telomere fusion patterns detected in the human reference genome are listed.

Reference genome	Chromosome	Start	End	Expanded start	Expanded end
Hg38	2	113602750	113603250	113597750	113608250
Hg38	9	128154000	128154750	128149000	128159750
Hg19	2	114360250	114360750	114355250	114365750
Hg19	9	130916250	130917000	130911250	130922000
T2T-CHM13	2	114027500	114028000	114022500	114033000
T2T-CHM13	9	140361250	140361750	140356250	140366750

Supplementary Table 2. Telomere fusion rates for the newly sequenced glioblastoma samples.

Sample	Donor ID	Donor No.	Sex	Age	Biopsy type	Coverage	Chr9 endogenous	Inward	Outward	TF total
PD53665a	PD53665	A14466	Female	60-70	Tissue	77.552	0.954	0.031	0.077	0.107
PD53665c	PD53665	A14466	Female	60-70	Blood	45.412	1.365	0.000	0.022	0.022
PD53665d	PD53665	A14466	Male	60-70	Serum	25.071	0.718	0.000	0.000	0.000
PD53670a	PD53670	A19294	Male	60-70	Tissue	86.655	1.154	0.052	0.062	0.113
PD53670c	PD53670	A19294	Male	60-70	Blood	47.803	1.632	0.000	0.000	0.000
PD53670d	PD53670	A19294	Male	60-70	Serum	30.467	0.853	0.000	0.000	0.000
PD54225a	PD54225	A10598	Male	40-50	Tissue	83.913	0.858	0.105	0.190	0.295
PD54225c	PD54225	A10598	Male	40-50	Serum	39.146	0.971	0.026	0.000	0.026
PD54225d	PD54225	A10598	Male	40-50	Blood	46.783	1.026	0.000	0.128	0.128
PD54226a	PD54226	A13852	Male	70-80	Tissue	82.904	1.061	0.000	0.101	0.101
PD54226c	PD54226	A13852	Male	70-80	Serum	27.173	0.957	0.037	0.037	0.074
PD54226d	PD54226	A13852	Male	70-80	Blood	47.305	1.226	0.021	0.000	0.021
PD54227a	PD54227	A14584	Male	70-80	Tissue	77.451	1.498	0.177	0.076	0.253
PD54227c	PD54227	A14584	Male	70-80	Serum	24.014	1.832	0.042	0.042	0.083
PD54227d	PD54227	A14584	Male	70-80	Blood	52.167	1.265	0.000	0.000	0.000
PD54229a	PD54229	A15045	Male	60-70	Tissue	85.610	0.934	0.000	0.052	0.052
PD54229c	PD54229	A15045	Male	60-70	Serum	27.578	0.798	0.000	0.036	0.036
PD54229d	PD54229	A15045	Male	60-70	Blood	41.341	1.258	0.024	0.024	0.048
PD54230a	PD54230	A16536	Male	60-70	Tissue	84.169	1.141	0.019	0.057	0.075
PD54230c	PD54230	A16536	Male	60-70	Serum	32.747	1.038	0.000	0.061	0.061
PD54230d	PD54230	A16536	Male	60-70	Blood	48.711	1.642	0.000	0.062	0.062
PD54231a	PD54231	A16590	Male	70-80	Tissue	83.627	1.411	0.039	0.176	0.216
PD54231c	PD54231	A16590	Male	70-80	Serum	14.580	2.332	0.000	0.000	0.000
PD54231d	PD54231	A16590	Male	70-80	Blood	47.957	1.043	0.000	0.000	0.000
