# Supplementary material for: The ALT pathway generates telomere fusions that can be detected in the blood of cancer patients
Source: Nat Commun. 2024 Jan 2;15:82. doi: 10.1038/s41467-023-44287-8 (PMC10762111; doi:10.1038/s41467-023-44287-8)
Supplement: Supplementary file 3 — Description of Additional Supplementary Files [file 41467_2023_44287_MOESM3_ESM.docx]

**Description of Additional Supplementary Files**

Supplementary Data 1

Description: Telomere fusion rates for all samples analysed.

Supplementary Data 2

Description: Analysis of the length of the fragments containing ALT-TFs. Significance was assessed using the Pearson's Chi-squared test and FDR correction.

Supplementary Data 3

Description: List of breakpoint sequences detected in pure ALT-TFs.

Supplementary Data 4

Description: List of 117 variables used to train the Random Forest models designed to predict the ALT status of tumours, and for the detection of cancer based on the features of the ALT-TFs detected in blood samples.

Supplementary Data 5

Description: Summary of the Random Forest model results used to predict cancer status.
