# Supplementary material for: The ALT pathway generates telomere fusions that can be detected in the blood of cancer patients
Source: Nat Commun. 2024 Jan 2;15:82. doi: 10.1038/s41467-023-44287-8 (PMC10762111; doi:10.1038/s41467-023-44287-8)
Supplement: Supplementary file 5 — Reporting Summary [file 41467_2023_44287_MOESM5_ESM.pdf]

nature portfolio

Corresponding author(s): Isidro Cortés-Cirino, PhD

Last updated by author(s): Oct 12, 2023

Reporting Summary

Nature Portfolio wishes to improve the reproducibility of the work that we publish. This form provides structure for consistency and transparency in reporting. For further information on Nature Portfolio policies, see our Editorial Policies and the Editorial Policy Checklist.

Statistics

For all statistical analyses, confirm that the following items are present in the figure legend, table legend, main text, or Methods section.

n/a	Confirmed	
☐	☒	The exact sample size (*n*) for each experimental group/condition, given as a discrete number and unit of measurement
☐	☒	A statement on whether measurements were taken from distinct samples or whether the same sample was measured repeatedly
☐	☒	The statistical test(s) used AND whether they are one- or two-sided<br>*Only common tests should be described solely by name; describe more complex techniques in the Methods section.*
☐	☒	A description of all covariates tested
☐	☒	A description of any assumptions or corrections, such as tests of normality and adjustment for multiple comparisons
☐	☒	A full description of the statistical parameters including central tendency (e.g. means) or other basic estimates (e.g. regression coefficient) AND variation (e.g. standard deviation) or associated estimates of uncertainty (e.g. confidence intervals)
☐	☒	For null hypothesis testing, the test statistic (e.g. *F*, *t*, *r*) with confidence intervals, effect sizes, degrees of freedom and *P* value noted<br>*Give P values as exact values whenever suitable.*
☒	☐	For Bayesian analysis, information on the choice of priors and Markov chain Monte Carlo settings
☒	☐	For hierarchical and complex designs, identification of the appropriate level for tests and full reporting of outcomes
☐	☒	Estimates of effect sizes (e.g. Cohen's *d*, Pearson's *r*), indicating how they were calculated

Our web collection on statistics for biologists contains articles on many of the points above.

Software and code

Policy information about availability of computer code

Data collection

The code to detect ATL-TFs using sequencing data is available at: https://github.com/cortes- ciriano-lab/telomere_fusions

Data analysis

We developed TelFusDetector to detect TFs using sequencing data (https://github.com/cortes-ciriano-lab/telomere_fusions). To identify regions containing fusion-like patterns in the human genome that could be misclassified as somatic, we first scanned the human reference genome (builds hg19, GRCh38 and T2T-CHM13) for patterns of TFs in windows of 500bp with an overlap of 250bp. As expected, this analysis yielded the relic of a telomere fusion in chromosome 2. In addition, we discovered additional regions with forward and reverse telomere repeats in chromosome 9, which we term "chr9 endogenous fusion". The coordinates for the genomic regions in the reference genome builds hg19, GRCh38 and T2T-CHM13 containing telomeric repeats are provided in Supplementary Table 1.
To detect somatic TFs in sequencing data sets, we extracted aligned sequencing reads containing at least two consecutive TTAGGG and two consecutive CCCTAA telomere sequences using custom python scripts relying on the Pysam and regex modules (https://github.com/pysam-developers/pysam). We allowed for up to two mismatches in the telomeric repeats to consider TVRs21. Next, we extracted the mate for each read containing telomeric repeats, and filtered out duplicate reads, as well as supplementary and complementary alignments. Reads mapping to regions in the reference genome containing telomere fusion-like patterns were discounted as somatic.
For the detection of TFs in ALaP-Seq data we used an alternative pipeline that applied FastQC to control for the quality of reads, Cutadapt to remove adapter sequences, and in-house Perl scripts. First, we identified candidate reads containing putative fusions by searching for the presence of two consecutive circularly permuted TTAGGG motifs and two consecutive, circularly permuted CCCTAA motifs. Reads mapping to endogenous fusions identified in the mouse reference genome (mm10) were discarded. Finally, we classified the ALT-TFs by analysing the relative orientation of the circularly permuted TTAGGG and CCCTAA repeats and the length of the breakpoint sequence.

Other software used in this study are: BWA-mem v0.7.17, R v4.3.1, wgsim v1.11, EMBOSS Merger v6.6.0.0, R packages RandomForest v4.7.1.1 and HMMcopy v0.99.0, ichorCNA v0.3.2, SAMtools v1.11, Python v3.7.7, and Python libraries regex v2.5.123 and Pysam v0.15.3.

For manuscripts utilizing custom algorithms or software that are central to the research but not yet described in published literature, software must be made available to editors and reviewers. We strongly encourage code deposition in a community repository (e.g. GitHub). See the Nature Portfolio guidelines for submitting code & software for further information.

Data

Policy information about availability of data

All manuscripts must include a data availability statement. This statement should provide the following information, where applicable:

- Accession codes, unique identifiers, or web links for publicly available datasets
- A description of any restrictions on data availability
- For clinical datasets or third party data, please ensure that the statement adheres to our policy

The raw sequencing data from the PCAWG project is available through controlled access application to the International Cancer Genome Consortium Data Access Compliance Office (DACO; http://icgc.org/daco) for the ICGC portion, and to the TCGA Data Access Committee (DAC) via dbGaP for the TCGA portion (https://dbgap.ncbi.nlm.nih.gov/aa/wga.cgi?page=login; dbGaP Study Accession: phs000178.v1.p1). Additional information on accessing the PCAWG data, including processed al. and an equal number of non-ALT samples from cancer patients to the training set, and held out the rest for testing the model. We encoded the ALT-TFs detected in each sample using a set of 117 variables, which are listed in Supplementary Table 6. and Sieverling et al. We then randomly assigned two-thirds of the ALT-positive samples data sets, can be found at https://docs.icgc.org/pcawg/data/. We used the following data sets from PCAWG, which are available at Synapse (https://www.synapse.org/) under the Synapse IDs syn10389158: clinical data from each patient, including tumour stage and vital status; syn1038916: harmonised tumour histopathology annotations using a standardised hierarchical ontology; and syn8272483: purity and ploidy values for each tumour sample. The raw sequencing data generated by the GTEx project is available through controlled access application via dbGAP (dbGaP Study Accession: phs000424.v8.p2). The raw sequencing data generated by the Genetic Epidemiology of COPD (COPDGene) project, which is part of TOPMed, is available through controlled access application via dbGAP (dbGaP Study Accession: phs000951.v4.p4). The raw sequencing data from the Korean Personal Genome Project (KPGP) were downloaded from SRA (study accession: PRJNA284338). Raw sequencing data from the CPTAC3 study is available through controlled access application to the NCI Data Access Committee (DAC) via dbGAP (dbGaP Study Accession: phs001287.v5.p4). WGS data from 124 retinal pigment epithelium (RPE) clones were downloaded from the European Nucleotide Archive database under primary accession number PRJEB23723 10 and European Genome-Phenome Archive (EGA, hosted by the EBI and the CRG) under the accession number EGAD00001001629 . The raw sequencing data from the Panbody project were downloaded from EGA (EGAD00001006641). PacBio and Illumina WGS data from the breast cancer cell line SK-BR-3 were downloaded from http://schatz-lab.org/publications/SKBR3/ . Nanopore and Illumina WGS data from the melanoma COLO829 cell line were downloaded from the European Nucleotide Archive (ENA) under the study accession PRJEB27698 26 PacBio WGS data from the colon cancer cell lines HCT116, KM12, SW620 and SW837 are available at the Gene Expression Omnibus (GEO) database under the accession number GSE149709 . AlaP-Seq and CHIRT-seq data are available at GEO under accession numbers GSE135563 and GSE79180, respectively. We used the builds of the human reference genome hg19, GRCh38 and T2T-CHM13. The raw sequencing data from the newly sequenced glioblastoma samples are available under controlled access at EGA under the accession number EGAD00001012101 [https://ega-archive.org/datasets/EGAD00001012101].

Human research participants

Policy information about studies involving human research participants and Sex and Gender in Research.

Reporting on sex and gender	Sex information was collected from the publications from which the data were obtained.
Population characteristics	We collected the following information from glioblastoma patients: Age, biological sex (male or female), and Biopsy_type (tumour tissue, whole blood or serum).
Recruitment	We were not involved in the design of the recruitment procedure for the sequencing data sets analysed, except for the glioblastoma samples newly sequenced for this study. Glioblatoma patients were enrolled by C.C.C. at the Neurosurgery Department at Centro Hospitalar Universitário Lisboa Norte (CHULN). Glioblastoma patients whose samples were analysed in this study were not preselected based on ethnicity, sex, age or any other criteria beyond diagnosis of glioblastoma.
Ethics oversight	The glioblastoma and blood samples used in this study were collected at the Neurosurgery Department at Centro Hospitalar Universitário Lisboa Norte (CHULN) and stored at Biobanco- iMM CAML (Lisbon Academic Medical Center, Lisbon, Portugal).| Ethical approval was obtained from the Ethics Committee of CHULN (Ref. No 367/18). Written informed consent was obtained from all patients prior to study participation in accordance with the European and National Ethical Regulation (law 12/2005). Patients did not receive financial compensation for donating samples. Data collection was carried out by a health care professional and included age and self-reported sex information. Surgical samples, not needed for diagnostic purposes, were processed less than 1 hour after surgery and included in the brain tumour collection of Biobanco-iMM CAML.

Note that full information on the approval of the study protocol must also be provided in the manuscript.

Field-specific reporting

Please select the one below that is the best fit for your research. If you are not sure, read the appropriate sections before making your selection.

☒ Life sciences ☐ Behavioural & social sciences ☐ Ecological, evolutionary & environmental sciences

For a reference copy of the document with all sections, see nature.com/documents/nr-reporting-summary-flat.pdf

Life sciences study design

All studies must disclose on these points even when the disclosure is negative.

Sample size	No sample size calculation was performed. Sample size was determined by availability of material of sufficient quality for whole-genome sequencing.
Data exclusions	No data points were excluded.
Replication	We performed whole-genome sequencing of 8 glioblastomas for external validation. The results identified in the PCAWG analysis were also replicated in other data sets, such as TARGET and CCLE.
Randomization	Randomization was applied for the training of the machine learning models. No other randomization was performed.
Blinding	Blinding was not performed as the individual whose samples we analysed are patients of C.C.C

Reporting for specific materials, systems and methods

We require information from authors about some types of materials, experimental systems and methods used in many studies. Here, indicate whether each material, system or method listed is relevant to your study. If you are not sure if a list item applies to your research, read the appropriate section before selecting a response.

Materials & experimental systems

n/a	Involved in the study	
☒	☐	Antibodies
☒	☐	Eukaryotic cell lines
☒	☐	Palaeontology and archaeology
☒	☐	Animals and other organisms
☐	☒	Clinical data
☒	☐	Dual use research of concern

Methods

n/a	Involved in the study	
☒	☐	ChIP-seq
☒	☐	Flow cytometry
☒	☐	MRI-based neuroimaging

Clinical data

Policy information about clinical studies

All manuscripts should comply with the ICMJE guidelines for publication of clinical research and a completed CONSORT checklist must be included with all submissions.

Clinical trial registration	The glioblastoma and blood samples used in this study were collected in the Neurosurgery Department at Centro Hospitalar Universitário Lisboa Norte (CHULN) and stored at Biobanco-iMM CAML (Lisbon Academic Medical Center, Lisbon, Portugal) in accordance with the Ethics Committee of CHULN (Ref. Nº 367/18).
Study protocol	The glioblastoma and blood samples used in this study were collected in the Neurosurgery Department at Centro Hospitalar Universitário Lisboa Norte (CHULN) and stored at Biobanco-iMM CAML (Lisbon Academic Medical Center, Lisbon, Portugal) in accordance with the Ethics Committee of CHULN (Ref. Nº 367/18). Written informed consent was obtained from all patients prior to study participation in accordance with the European and National Ethical Regulation (law 12/2005).
Data collection	Data collection was carried out by a health care professional and included detailed clinical information such as age, gender, and EGFR mutational status. Surgical samples, not needed for diagnostic purposes, were processed less than 1 hour after surgery and included in the brain tumour collection of Biobanco-iMM CAML.
Outcomes	No outcome data was considered.

March 2021
